# Characteristics of the environment and physical activity in midlife: Findings from UK Biobank

**DOI:** 10.1016/j.ypmed.2018.10.024

**Published:** 2019-01

**Authors:** Lindsey Smith, Jenna Panter, David Ogilvie

**Affiliations:** MRC Epidemiology Unit & UKCRC Centre for Diet and Activity Research (CEDAR), University of Cambridge, School of Clinical Medicine, Box 285, Cambridge Biomedical Campus, Cambridge, Cambridgeshire CB2 0QQ, UK

**Keywords:** Environment, Physical activity, Accelerometry, Walking, UK Biobank

## Abstract

Characteristics of the environment influence health and may promote physical activity. We explored the associations between neighborhood environmental characteristics grouped within five facets (spaces for physical activity, walkability, disturbance, natural environment, and the sociodemographic environment) and objective (‘recorded’) and self-reported (‘reported’) physical activity in adults from UK Biobank. Recorded activity was assessed using wrist-worn accelerometers (2013–2015, *n* = 65,967) and time spent in moderate-to-vigorous physical activity (MVPA), walking, and walking for pleasure was self-reported (2006–2010, *n* = 337,822). Associations were assessed using linear and multinomial logistic regression models and data were analyzed in 2017. We found participants living in areas with higher concentrations of air pollution recorded and reported lower levels of physical activity and those in rural areas and more walkable areas had higher levels of both recorded and reported activity. Some associations varied according to the specificity of the outcome, for example, those living in the most deprived areas were less likely to record higher levels of MVPA (upper tertile: RRR: 0.80 95% CI: 0.74, 0.86) but were more likely to report higher levels of walking (upper tertile: RRR: 1.09, 95% CI: 1.06, 1.13). Environmental characteristics have the potential to contribute to different physical activities but interventions which focus on a single environmental attribute or physical activity outcome may not have the greatest benefits.

## Highlights

• Environmental characteristics of varying scales were considered in combination.
• Physical activity outcomes were measured using recorded and reported assessments.
• Associations for each characteristic varied according to specificity of outcome.
• Interventions targeting single characteristics may have trade-offs for health and place.

## 1 Introduction

Physical inactivity accounts for 9% of premature mortality worldwide and engaging in regular physical activity reduces the risk of non-communicable diseases including cardiovascular disease, type 2 diabetes, and some cancers (Lee et al., 2012). Moderate-to-vigorous physical activity (MVPA) confers health benefits and allows for comparisons with activity recommendations (World Health Organization, 2010). Activities such as walking could also foster social interactions, promote social equity, improve air quality, and lead to more environmentally sustainable communities by displacing car use (Giles-Corti et al., 2010; Hunter et al., 2015). However, many adults do not achieve sufficient levels of activity (World Health Organization, 2016).

It is hypothesized that the environment and social context in which people live is related to physical activity (Sallis et al., 2006). The number of studies exploring these associations has increased in the past 20 years with much of the literature focused on micro-level attributes of the physical built environment which may provide spaces for use and improve destination accessibility (Bauman et al., 2012; Giles-Corti et al., 2016; Van Holle et al., 2012).

Applying a public health perspective and embracing the notion of the wider social determinants of health (Dahlgren and Whitehead, 2006; Giles-Corti et al., 2016) suggests a range of micro- and macro-level environmental attributes might be important. Contextual conditions such as deprivation and rurality are likely to influence health behaviors, as well as more immediate conditions of environmental disturbance or the natural environment which affect the desirability to use space. It is postulated that high levels of pollutants increase the perception of risk (Hankey et al., 2012) and discourage outdoor activity. Additionally, poorer communities are often disproportionally exposed to air pollution (Hajat et al., 2015). Few studies have examined the role of air pollution and its association with physical activity (An et al., 2017) and none have assessed contextual characteristics, such as deprivation and air pollution, and micro-level characteristics of urban form. Investigating these simultaneously may help provide a broader perspective on the role of the residential environment as it relates to physical activity. This is important for better understanding the trade-offs between characteristics more or less conducive to physical activity and the implications for public health.

Objective measures enable precise data to be collected on duration and intensity of activity (Esliger and Tremblay, 2007). A large-scale study of participants living in 14 cities found that parks and greater residential density in the neighborhood were positively associated with objectively measured MVPA (Sallis et al., 2016), however, specific behaviors were not investigated. The most consistent associations are drawn from studies where domain or activity-specific outcomes and exposure measures are well-matched (Bauman et al., 2012). For example, a UK study found that greenness was associated with active commuting and walking which contribute to overall MVPA (Sarkar, 2017). However, it is difficult to identify these activities accurately from objective physical activity data alone. Combining objective with self-reported measures of activities such as walking can therefore complement precise estimates of total activity with information on specific activity behaviors.

Using a large dataset with geographical heterogeneity, we aim to assess the associations between environmental characteristics in the residential neighborhood and a range of objective (‘recorded’) and self-reported (‘reported’) measures of physical activity and walking. Characteristics are described under five broad facets (spaces for physical activity, walkability, disturbance, the natural environment, and the sociodemographic environment) which range from micro-level environments considered to encourage specific types of activity, to macro-level environments which may affect levels of activity more generally. Physical activity measures increase in specificity from recorded total activity to reported time in walking behaviors.

## 2 Methods

### 2.1 Study design

Cross-sectional data were used from the UK Biobank study, collected from 502,656 participants aged 37–73 years at recruitment. Respondents were invited if they were registered with the National Health Service (NHS) and lived within 35 km of one of 22 Biobank assessment centers. Baseline data including sociodemographic, lifestyle, and physical activity information were self-reported between March 2006 and July 2010 (UK Biobank, 2006). A random sub-sample of participants (*n* = 236,519) who provided a valid email address were invited to take part in objective physical activity measures (Doherty et al., 2017). Accelerometers (Axivity AX3) were posted to those who agreed to participate (44.8%, *n* = 106,053) and worn on their dominant wrist continuously for seven days (Doherty et al., 2017) between June 2013 and December 2015. Data from wrist worn devices have been validated against established measures of physical activity energy expenditure (White et al., 2016).

The UK Biobank study has ethical approval from the North West Multi-center Research Ethics Committee (MREC), Information Advisory Group (IAG), and the Community Health Index Advisory Group (CHIAG). Details on the Biobank study design and survey methods are described in a full protocol and accompanying paper (UK Biobank, 2010, UK Biobank, 2007).

### 2.2 Inclusion criteria

We restricted our analysis to participants who had data on environmental characteristics, covariates and at least one physical activity outcome. Sub-samples of participants were followed up (*n* = 20,346 December 2009–June 2013 and *n* = 11,923 April 2014–November 2016). If any of these participants indicated they had moved home, they were excluded from the analysis (Supplemental File 1).

### 2.3 Physical activity

Five physical activity outcomes were included for analysis based on the accuracy and specificity of the measure.

### 2.4 Recorded physical activity

Two measures of physical activity were derived: mean acceleration, which assesses average volume of activity in milli-gravity units (m*g*), and time spent in MVPA (Supplemental File 1) which equates to 134 m*g* of acceleration captured by the dominant wrist (White et al., 2016). We computed the total minutes spent above 125 m*g*, which was the closest available threshold in the processed data.

### 2.5 Reported physical activity

In relation to MVPA and total walking, participants were asked how many days in a typical week they did each type of activity for at least 10 min and the duration of each episode (Supplemental File 1). The number of reported days was multiplied by the duration to calculate the weekly time spent in each activity. Walking for pleasure was assessed in a similar way, except that categorical response items were used. Weekly time was derived by assigning the median number of times per week and durations.

### 2.6 Environmental data

The UK Biobank Urban Morphometric Platform (UKBUMP) is a nationwide resource and uses objective data to characterize environmental conditions that influence health using a range of buffer sizes around each participant's home location (Sarkar et al., 2015). Variables were based on a conceptual model (Sarkar et al., 2015) and derived to serve a range of research questions related to physical activity, diet, alcohol consumption and general health. The processes are described in detail elsewhere (Sarkar et al., 2015, Sarkar et al., 2014). Briefly, a measure of environmental conditions is available for each participant, based on the characteristics within a defined straight-line or network distance of their residential address. We used measures which characterized the area within 1 km, or closest available distance, as this corresponds to a 10–15 min walk, and 0.8–1 km is commonly used and broadly accepted in the literature (Bancroft et al., 2015; Mason et al., 2018).

We chose 15 variables conceptually and most plausibly related to physical activity (excluding those related to diet and alcohol consumption). These variables were grouped into five broad facets (spaces for physical activity, walkability, disturbance, natural environment, and the sociodemographic environment) based on theme and their influence on different activity types (Table 1).

**Table 1:** Description and classification of objectively measured environmental variables.

Variable^b^	Description	Spatial scale buffer type	Data source^a^, year	Classification
Spaces for physical activity				
Facilities for physical activity	Presence of facilities for physical activity	1km^c^ Network	UK OS AddressBase premium point data, 2013	No/yes
Parks	Presence of parks	1km^c^ Network	UK OS AddressBase premium point data, 2013	No/yes

Walkability				
Walkability	Composite measure of street connectivity, residential density and land use mix^b^ Z scores of component measures were generated and summed	n/a	Derived from UK OS ITN, 2010 and UK OS AddressBase premium point data, 2013	Quartile

Disturbance				
Air pollution	Annual average for concentration of nitrogen oxides (NO_X_)	Interpolated from model at residential address	European Study of Cohorts for Air Pollution Effects (ESCAPE) Land Use Regression model, 2010	<26 μgm^−3^/≥ 26 μgm^−3^
Noise pollution	Average daytime sound level pressure over 12-hour period (07:00 to 19:00)	Interpolated from model at residential address	Common Noise Assessment Methods (CNOSSOS-EU) model, 2009	<54 kHz/≥ 54 kHz
Distance to major road	Inverse distance to the nearest major road based upon a local road network where a major road is a road with traffic intensity >5000 motor vehicles per 24 h	n/a	Road network: OS Meridian 2 road network, 2009 Traffic data: Eurostreets (vs 3.1) digital road network, 2008	Quartile

Natural environment				
Terrain	Mean slope angle	1km^c^ Circular	Landmap DTM (5 m resolution) Stereo aerial photography 1998–2008	<3°/≥3°
Greenness	Mean normalized deviation vegetation index (NDVI)	0.5 km Circular	CIR Landmap satellite data (5 m resolution), 2006–2010	Quartile

Sociodemographic environment				
Urban-rural status	Based on population density	Postcode	Office for National Statistics Postcode Directory (ONSPD) and UK Census data, 2001	Urban/fringe/rural
Area-level deprivation	Townsend deprivation index	Census output area	UK Census data, 2001	Quintile

OS = Ordnance Survey; ITN = Integrated Transport Network; DTM = Digital Terrain Model; CIR = Color Infrared.

a For further details on data sources, please refer to UKBUMP data analysis and specification manual (Sarkar et al., 2014).

b For further details on the derivation of variables and component measures, please refer to Supplemental File 1.

c 0.5 km distance used for sensitivity analyses to investigate the effects of smaller neighborhood measures.

### 2.7 Covariates

All covariates were derived or self-reported in the lifestyle questionnaire during baseline assessment and comprised age, sex, ethnicity, assessment center, highest educational qualification, income, employment status, housing tenure, number of vehicles in household, whether children lived in the household, urban-rural status, and area-level deprivation.

### 2.8 Statistical analysis

Descriptive analyses were undertaken to assess the characteristics of included and excluded samples, and Wilcoxon rank sum tests were used to compare recorded and reported activity.

We used linear regression models to assess the associations between the environmental characteristics and mean acceleration and multinomial logistic regression models for tertiles of recorded and reported time spent in MVPA, walking, and walking for pleasure as preliminary analyses indicated that assumptions of linear regression could not be satisfied. First, univariate regression analyses were conducted for each environmental characteristic, adjusting for covariates (Model 0). All significant characteristics (*p* < 0.05) were carried forwards into a single adjusted model for each activity outcome (Model 1). We assessed significance with tests for trend across each activity tertile.

### 2.9 Sensitivity analysis

We ran sensitivity analyses to explore which components of the walkability scores (street connectivity, land use mix, and residential density) contributed most to any associations observed. To investigate the effects of using smaller neighborhood measures, further sensitivity analyses were performed by repeating the process with smaller distances for facilities for physical activity, parks, walkability, and terrain.

## 3 Results

### 3.1 Sample

**Fig. 1:**
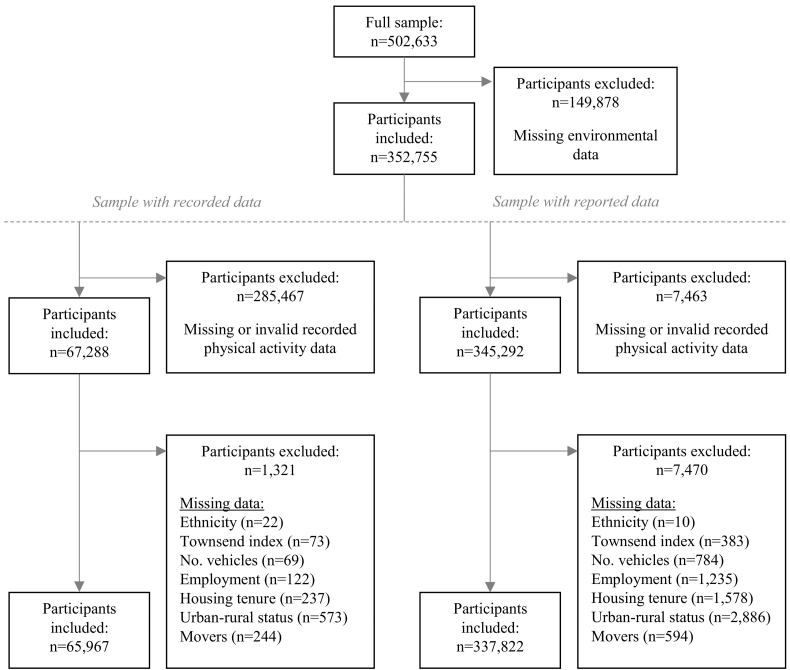
Flowchart of process for inclusion for participants with reported and recorded physical activity data.

Environmental data for all exposures of interest was available for 352,755 participants (70.2% of full sample), of whom 65,967 (18.7%) had valid recorded physical activity measures and 337,822 (95.8%) provided information on at least one of the three reported outcomes (Fig. 1). The distribution of characteristics was similar for all samples (Table 2). The sample with reported physical activity data were most similar to the full sample while the sample with recorded physical activity data contained a higher proportion of women and were more likely to be educated to degree level, in paid employment, a home owner, and have access to a vehicle.

**Table 2:** Sample characteristics.

	Full sample (*n* = 502,633)	Sample who had environmental data available (*n* = 352,755)	Sample who provided recorded physical activity data (*n* = 65,967)	Sample who provided reported physical activity data^a^ (*n* = 337,882)
	*n* (%)	*n* (%)	*n* (%)	*n* (%)
Sex				
Male	229,171 (45.6)	160,917 (45.6)	28,718 (43.5)	154,042 (45.6)
Female	273,462 (54.4)	191,838 (54.4)	37,249 (56.5)	183,780 (54.4)
Age at baseline				
40–49	117,903 (23.5)	82,573 (23.4)	15,282 (23.2)	78,590 (23.3)
50–59	167,191 (33.3)	116,173 (32.9)	23,686 (35.9)	111,438 (33)
60–69	215,112 (42.8)	152,292 (43.2)	26,767 (40.6)	146,145 (43.3)
70–79	2427 (0.5)	1717 (0.5)	232 (0.4)	1649 (0.5)
Age at recorded physical activity assessment				
40–49	8785 (8.5)	6331 (8.8)	5544 (8.4)	n/a
50–59	29,911 (28.8)	20,758 (28.8)	18,761 (28.4)	
60–69	45,938 (44.3)	31,887 (44.2)	29,388 (44.5)	
70–79	19,076 (18.3)	13,188 (18.3)	12,274 (18.6)	
Ethnicity				
White	472,816 (94.6)	331,981 (94.2)	63,689 (96.5)	319,543 (94.6)
Non-white	27,039 (5.4)	20,267 (5.8)	2278 (3.5)	18,279 (5.4)
Weight status				
Underweight/normal	165,073 (33.0)	114,334 (32.6)	25,556 (38.8)	110,381 (32.8)
Overweight	212,168 (42.5)	149,218 (42.6)	27,189 (41.3)	143,671 (42.7)
Obese	122,287 (24.5)	87,079 (24.8)	13,085 (19.9)	82,266 (24.5)
Urban-rural status				
Urban	428,890 (86.2)	303,764 (86.9)	56,059 (85.0)	293,056 (86.7)
Fringe	33,865 (6.8)	24,226 (6.9)	5050 (7.7)	23,613 (7.0)
Rural	34,803 (7.0)	21,676 (6.2)	4858 (7.4)	21,153 (6.3)
Highest educational qualification				
College or university degree	161,206 (32.4)	109,644 (31.1)	27,666 (41.9)	106,575 (31.5)
Other professional (e.g. teaching)	25,810 (5.2)	18,328 (5.2)	3365 (5.1)	17,623 (5.2)
Higher education (e.g. A Levels, NVQ)	88,070 (17.7)	61,692 (17.5)	12,170 (18.4)	59,627 (17.7)
Secondary education (e.g. GCSEs)	132,113 (26.5)	97,224 (27.6)	16,794 (25.5)	93,810 (27.8)
Other	90,787 (18.2)	65,381 (18.6)	5972 (9.1)	60,187 (17.8)
Employment status				
Paid employment or self-employment	287,225 (57.2)	199,930 (56.8)	40,229 (61.0)	193,972 (57.4)
Retired	167,013 (33.3)	118,909 (33.8)	21,171 (32.1)	114,604 (33.9)
Unable to work	16,836 (3.4)	12,009 (3.4)	1123 (1.7)	10,408 (3.1)
Unemployed	8265 (1.6)	5880 (1.7)	780 (1.2)	5481 (1.6)
Home duties, carer, student, volunteer, or other	22,423 (4.5)	15,541 (4.4)	2664 (4.0)	13,357 (4.0)
Housing tenure				
Home owner	442,566 (89.6)	312,526 (88.9)	62,232 (94.3)	304,046 (90.0)
Renting	46,462 (9.4)	31,452 (8.9)	3066 (4.6)	28,747 (8.5)
Other	5123 (1.0)	7449 (2.1)	669 (1.0)	5029 (1.5)
No. vehicles in household				
Two or more	245,129 (49.0)	170,355 (48.5)	34,839 (52.8)	165,238 (48.9)
One	208,636 (41.7)	149,192 (42.5)	27,420 (41.6)	143,131 (42.4)
Other	46,606 (9.3)	31,878 (9.1)	3708 (5.6)	29,453 (8.7)
People in the household				
One	92,942 (18.6)	63,395 (18.1)	10,691 (16.2)	60,478 (18.0)
Two	232,811 (46.6)	164,856 (47.1)	31,655 (48.1)	159,104 (47.2)
Three or more	172,324 (34.5)	121,638 (34.8)	23,527 (35.7)	117,178 (34.8)
Children in household				
No	324,331 (64.8)	227,131 (64.6)	41,756 (63.3)	217,580 (64.4)
Yes	176,040 (35.2)	124,294 (35.4)	24,211 (36.7)	120,242 (35.6)

a This sample included any participant who provided information on any of the three reported outcomes (time spent in MVPA, total walking, or walking for pleasure).

### 3.2 MVPA, total walking and walking for pleasure

For each tertile of recorded MVPA, the greatest proportion of participants was in the corresponding tertile of reported MVPA (Table 3, Panel A). Similar and more convincing patterns are shown for reported MVPA and walking (Panel B), and walking and walking for pleasure (Panel C). Tests for trend indicated each pair of measures were related (*p* < 0.001).

**Table 3:** Comparing reported and recorded physical activity and walking behaviors.

		Lower tertile n (%)	Middle tertile n (%)	Upper tertile n (%)
Panel A:				
		**Recorded time spent in MVPA**		
Reported time spent in MVPA	Lower tertile	8357 (39)	7000 (32)	5102 (33)
	Middle tertile	7337 (34)	8095 (37)	7868 (36)
	Upper tertile	5892 (27)	6926 (31)	8856 (41)
	Total	21,586 (100)	22,021 (100)	21,826 (100)

Panel B:				
		**Reported time spent in MVPA**		
Reported time spent walking	Lower tertile	57,467 (52)	39,264 (35)	18,814 (16)
	Middle tertile	33,723 (30)	43,012 (38)	33,199 (29)
	Upper tertile	20,057 (18)	29,984 (27)	62,302 (55)
	Total	111,247 (100)	112,260 (100)	114,315 (100)

Panel C:				
		**Reported time spent in walking**		
Reported time spent walking for pleasure	Lower tertile	58,570 (51)	33,460 (30)	30,647 (27)
	Middle tertile	39,467 (34)	34,658 (32)	26,996 (24)
	Upper tertile	17,508 (15)	41,816 (38)	54,700 (49)
	Total	115,545 (100)	109,934 (100)	112,343 (100)

Panel A: Percentages given are of participants in reported MVPA strata for recorded MVPA tertile.

Panel B: Percentages given are of participants in reported time spent walking strata for reported MVPA tertile.

Panel C: Percentages given are of participants in reported time spent walking for pleasure strata for reported total walking tertile.

### 3.3 Associations between environmental characteristics and physical activity

**Fig. 2:**
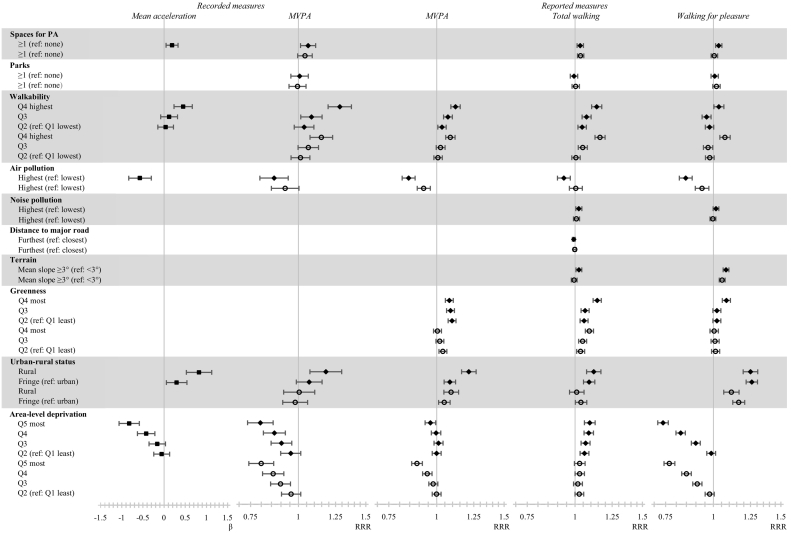
Adjusted associations between environmental characteristics and activity outcomes (Model 1) Outcome variables: ■ Continuous data; ♦ Upper tertile; ○ Middle tertile;  95% Confidence interval. White space is where variables have not been included in Model 1 β = regression coefficient presented on linear scale; RRR = relative risk ratio presented on log scale; MVPA = moderate-to-vigorous physical activity.

Associations between environmental characteristics and physical activity were broadly similar in terms of magnitude and statistical significance between Model 0 and Model 1. We therefore present and discuss the results from Model 1 (Fig. 2 and Supplemental File 2, Table S1).

### 3.4 Spaces for physical activity

Access to facilities for activity was associated with higher mean acceleration (β: 0.19, 95% CI: 0.05, 0.33), higher levels of MVPA (upper tertile RRR: 1.06, 95% CI: 1.01, 1.11), total walking and walking for pleasure. Participants with access to a park, compared to those without, were more likely to report higher levels of walking for pleasure (middle tertile RRR: 1.02, 95% CI 1.00, 1.04).

### 3.5 Characteristics of walkability

Neighborhood walkability was associated with higher levels of reported and recorded activity (all *p* < 0.001), except for the upper tertile of walking for pleasure. When comparing the most walkable neighborhoods with the least, associations were largest for recorded MVPA (upper tertile RRR: 1.28, 95% CI 1.20, 1.38) and total walking (upper tertile RRR: 1.14, 95% CI 1.10, 1.17).

### 3.6 Characteristics of disturbance

Participants living in areas with highest concentrations of air pollution recorded a lower mean acceleration

(β: −0.57, 95% CI: −0.84, −0.30). The direction and magnitude of the association were consistent across all other outcomes with a weaker association for total walking. Those living in areas with highest levels of noise pollution were more likely to report higher levels of walking (upper tertile RRR: 1.02, 95% CI: 1.00, 1.04) than those in areas with lowest noise pollution. No significant associations were shown for distance to the nearest major road.

### 3.7 Characteristics of the natural environment

Participants living in areas with steepest terrain were more likely to report higher levels of walking (upper tertile: RRR 1.02, 95% CI: 1.01, 1.04) and walking for pleasure (upper tertile RRR: 1.08. 95% CI: 1.06, 1.10). Greener neighborhoods were generally associated with higher reported levels of MVPA, walking and walking for pleasure (*p* < 0.001).

### 3.8 Sociodemographic characteristics

Clear dose-response relationships were shown for characteristics of the sociodemographic environment and all activity outcomes. Participants living in rural areas typically recorded and reported higher levels of activity. Compared to urban dwellers, those in rural areas were more likely to report higher levels of walking for pleasure (upper tertile RRR: 1.25, 95% CI: 1.20, 1.31) which appears to explain the association shown for reported MVPA. Compared to those living in less deprived areas, participants in more deprived areas were less likely to record and report higher levels of activity and walking for pleasure. Findings for total walking were in the opposite direction.

### 3.9 Sensitivity analyses

Results for the individual walkability components indicated that land use mix was the biggest driver of these associations (Supplemental File 2, Table S2). For MVPA, measures of street connectivity appeared to be important, as did residential density for total walking and walking for pleasure.

The results of the adjusted models using smaller distances for facilities for physical activity, parks, walkability, and terrain indicated findings were qualitatively consistent with the original analysis (Supplemental File 2, Fig. S1).

## 4 Discussion

### 4.1 Principal findings

The study showed that characteristics of the neighborhood environment were associated with recorded and reported physical activity in a large UK sample of adults. Walkability, disturbance, and the sociodemographic characteristics showed the strongest associations with physical activity, even after adjusting for other characteristics. There were some differences between the associations observed for global measures of activity and more specific behaviors. For example, associations between walkability appeared stronger for total walking than walking for pleasure.

### 4.2 Comparisons with existing evidence

Our findings were generally consistent with previous research (Bauman et al., 2012; McCormack and Shiell, 2011; Van Holle et al., 2012) but some differences could be attributed to the methods used to assess outcomes and exposures or the characteristics of the sample.

The associations for access to facilities for physical activity were most strongly associated with total walking and walking for pleasure and access to parks was weakly associated with walking for pleasure. Mixed findings have been shown for different activity outcomes in the literature (Bancroft et al., 2015; McCormack and Shiell, 2011; Van Cauwenberg et al., 2011). Our study focused on physical proximity to facilities whereas others consider convenience, satisfaction and availability but tend not to give a detailed breakdown of the facilities under consideration (Bauman et al., 2012; McCormack and Shiell, 2011; Van Cauwenberg et al., 2011; Wendel-Vos et al., 2007). When our analyses were re-run to include a broader range of recreational facilities not designed specifically for activity (e.g. church halls) the results were not attenuated (data not shown). The weak associations for parks may be because neighborhood parks are not always the destination for physical activity, or that previous studies explored the size, perceived accessibility or quality of parks (Bancroft et al., 2015; McCormack and Shiell, 2011; Van Cauwenberg et al., 2011). By simultaneously including measures of disturbance and greenness in our analysis, we go some way towards accounting for this. Further studies could investigate the role of factors that moderate the associations between environmental characteristics and activity, such as quality of the environment (James et al., 2017).

We found strong positive associations with walkability and mean acceleration, MVPA and walking which is consistent with the literature (Bauman et al., 2012; McCormack and Shiell, 2011; Van Holle et al., 2012). Land use mix contributed most to the positive associations and this is recognized as an important determinant of total physical activity, MVPA, and walking (McCormack and Shiell, 2011; Van Holle et al., 2012). Greater residential density may be important for MVPA and walking, but this could be dependent on the availability of other land uses in the neighborhood, such as places to walk for pleasure. In contrast, while street connectivity may facilitate walking, connectivity alone may be less important for increasing levels of activity.

Our findings for disturbance of the environment showed that those living in more polluted areas were less likely to record or report higher levels of MVPA and walking for pleasure but the associations were less consistent for total walking. These findings may be attributed to walking for transport which often takes place in inner city areas where walkability is high but concentrations of particulate matter are also highest (Hankey et al., 2012; Marshall et al., 2009). Although we use a relatively coarse measure of annual NO_X_, few other studies have assessed the relationship between air pollution and physical activity. There is some evidence that exposure to air pollution may discourage other activities such as walking for pleasure (An and Xiang, 2015) which is consistent with our findings for urban-rural status.

Greenness was associated with reported but not recorded activity. Although the number of studies using both objective measures of physical activity and greenness is limited, one other study found strong non-linear associations (James et al., 2017). Those authors concluded that the greenness-physical activity relationship was weakened in areas of high walkability which may explain the lack of associations in our study.

Most of the literature on environmental associations of physical activity is from the USA or other areas of Europe (Bauman et al., 2012; McCormack and Shiell, 2011; Van Holle et al., 2012) and so the differences between our findings and previously published work may be due to differences in settings or the prevalence of baseline behaviors. Contradictory to current research (Van Holle et al., 2012; McCormack and Shiell, 2011), our study suggests those living in more rural areas report higher levels of walking for pleasure, even after adjusting for area-level deprivation and income. Participants who lived in more deprived areas generally recorded lower levels of activity, however, the same group were more likely to report the highest levels of total walking, possibly having done so out of necessity rather than choice. Measures of income and area-level deprivation may not have completely explained these differences and our results may be due to self-selection or preferences. For example, we found associations between steep terrain and walking for pleasure. As hilliness has rarely been assessed in the literature before, there are inconsistencies about the direction of association with different domains of activity (Van Holle et al., 2012; Wendel-Vos et al., 2007). Although we cannot be certain why and where activity takes place, one possible explanation could be that participants with a preference for walking choose to live in hillier neighborhoods or that activity in greener or hillier areas may be perceived to be longer due to aesthetics or a greater exertion of energy (Sun et al., 2015). This area warrants further investigation.

### 4.3 Strengths and limitations

The key strengths of the study were the large sample size and the combination of objective and self-reported measures of activity which allowed us to examine and compare different environmental associations for global and specific outcomes. While similar studies have used objective physical activity data from multiple countries (Sallis et al., 2016), they use data from one locality within each country and focus on a single outcome. We used geographically heterogeneous data from across the UK. Recognizing the importance of understanding a range of place-based determinants of health, we included immediate and contextual characteristics of the residential neighborhood, organized around five facets important for physical activity and public health. We were therefore able to examine environmental characteristics simultaneously and control for potential confounders.

Limitations of our study include the use of cross-sectional data meaning that we cannot make causal inferences and there is a risk of reverse causation. Although the sample is uniquely large and heterogeneous (UK Biobank, 2017), the included sample contained a high proportion of urban dwellers, homeowners, and participants educated to degree level which may be indicative of a volunteer or self-selection bias. More active participants may have chosen to participate in objective monitoring or to live in environments matched to their preferences for activity. Unfortunately, we had no further information on this.

Measures of the environment were limited to static neighborhood exposures. As there were no data available to locate physical activity or to describe environmental characteristics around other daily anchor points, such as the workplace, it was not possible to capture exposure to environments outside of the neighborhood where participants may be active. These unmeasured exposures may lead to residual confounding (Burgoine and Monsivais, 2013). Using previously-derived data also meant that the accuracy of the underlying data is unknown and there is a temporal and spatial mismatch across variables. However, the categorization of exposures helps to minimize the risk of misclassification and the sensitivity analyses showed the size of the neighborhood investigated made little difference to the pattern of findings.

The analyses of recorded and reported activity were not contemporaneous and used two different samples. Despite differences in age at times of assessment, the proportion of the samples employed and in retirement is similar which suggests the samples are comparable. To ensure characteristics of the neighborhood were classified correctly at the time of assessment, where information was available we removed participants who had moved home. As the number of movers was small, it is likely that the effect of any misclassification will be minimal. This information was not available for the entire cohort, but will be in time.

### 4.4 Future research

Further investigation into activity domains and behaviors in relation to a range of environmental characteristics is required. Applying methods to identify specific activity behaviors from objective data will allow for these relationships to be explored further and with more confidence. The use of large-scale GPS data will also enable assessment of exposures and activity locations within and outside the neighborhood. Combining objective measures with qualitative evidence on perceptions of space, such as aesthetics and safety, is also important for understanding how and why environments are used for physical activities. Lastly, longitudinal study designs are encouraged to understand how changes in the environment impact physical activity and to advance the field and guide interventions.

### 4.5 Policy implications

Modifying attributes of the physical environment may promote changes in physical activity. However, the evidence highlights the potential complexity in designing neighborhoods to support physical activity and encourage wider health benefits. Our study is one of the first to investigate air pollution in relation to reported and recorded physical activity. In doing so we see that while walkable neighborhoods may encourage activity, particularly total walking, higher levels of walking are associated with participants living in areas with higher concentrations of air pollution and in more deprived areas. Consequently, an environment conducive to walking may not have the greatest overall benefit for physical activity or health given the adverse effects of greater exposure to air pollution and social inequalities. While modifying neighborhoods to support physical activity may ultimately lead to sustained population changes, interventions which focus on a single characteristic of the environment or physical activity outcome are unlikely to have the greatest benefits. Instead, we recommend that comprehensive strategies be employed to address a range of environmental characteristics in combination with careful consideration of the trade-offs for people and places.

## Appendix A Supplementary data

Supplemental File 1 Additional methodological information Image 2

Supplemental File 2 Additional results Image 3
