# Supplementary material for: Characteristics of the environment and physical activity in midlife: Findings from UK Biobank
Source: Prev Med. 2019 Jan;118:150–8. doi: 10.1016/j.ypmed.2018.10.024 (PMC6344227; doi:10.1016/j.ypmed.2018.10.024)
Supplement: Supplementary file 1 — Supplemental File 1 Additional methodological information [file mmc1.docx]

**Supplemental File 1: Additional methodological information**

**Inclusion criteria**

Our analyses required participants to have data for all environmental variables and either objective or self-reported measures of physical activity. We therefore created two samples. Those with accelerometer data were included in the sample for recorded physical activity and those who provided information on either time spent in MVPA, total walking, or walking for pleasure were included in the second sample for reported activity. Participants were excluded from both samples if they had invalid or missing data for covariates. However, those who answered ‘do not know’ or ‘prefer not to answer’ for information on covariates were included for analysis to maintain the highest possible sample size.

We also considered excluding those whose mobility, and therefore physical activity, was limited. However, the data available on the presence of pain in the leg or chest when walking was only available for a sub-sample of participants and did not differentiate between musculoskeletal and cardiovascular issues. With this limited information, it is difficult to understand the nature of confounding clearly. Although it may be more difficult to walk, increased walking may be part of a rehabilitation program or self-selection may occur whereby participants live closer to facilities for ease of access or where reliance on private transport is easier. We therefore chose not to exclude participants from the sample if they had reported pain.

**Residential relocation**

As the recorded measures of physical activity were undertaken in a sub-sample after baseline, we assessed whether the measures of environmental exposures were still appropriate. Environmental exposure data came from a range of sources, including data from 2009 and 2010 which was the latter stages of time when self-reported physical activity data was available. To ensure that the environmental exposures were correctly classified, we needed information on residential location over the duration of the study period. Information on home location was collected at baseline for the entire sample and for two further time points for a sub-sample; firstly between December 2009 and June 2013 (n=20,346) and secondly between April 2014 and November 2014 (n=11,923). To identify those who moved, we compared the locations of residential addresses between baseline and follow-up. At the time of analysis, the scale at which coordinates were presented were different for both time points so the follow-up data was rounded to match the coarser scale used at baseline. If follow-up coordinates were different to baseline and there was assumed to be no rounding error, participants were classified as movers and excluded from analysis. As follow-up data was not available for all participants, it is unlikely all movers are captured but this is the most reasonable approach given the data available.

**Recorded physical activity**

Objectively-measured physical activity data were collected for a random sub-sample of participants from all assessment centers except those in the North West of England, which were excluded due to concerns of participant burden from trialing other new projects in this region.

Participants wore an accelerometer on their dominant wrist for seven days, including nighttime. Doherty and colleagues describe the calibration and data processing in detail (Doherty et al., 2017). Briefly, measures of acceleration were collected in five-second epochs, maintaining the mean acceleration over the duration of the epoch. The percentage of time spent in different ranges of acceleration for the week are available in the dataset. Given the fractional measures of acceleration are derived from a cumulative distribution function of all five second epochs, sustained bouts of acceleration are not accounted for in the data.

Non-wear time was previously identified as stationary episodes of at least 60 minutes and removed from the data. Only participants with more than 72 hours of wear time were included in the sample and periods of non-wear time had been imputed using the average magnitude from a similar time on a different day of measurement. A value of mean acceleration for the week was available in the dataset, calculated by averaging worn and imputed values. We used this value to assess the average volume of activity and to indicate a global measure of activity for the week.

MVPA equates to 3 METs which is equal to and acceleration of 134 m*g* for accelerometer data collected on the dominant wrist (White et al., 2016). To measure physical activity at a specific intensity, we used the fraction of time spent above the closest available acceleration threshold in the dataset (125m*g*) to estimate the total minutes spent in MVPA over the course of the week. The total time spent in MVPA was then divided into tertiles. Previous studies have used daily measures of bouted MVPA based on established cut-points of counts per minutes collected from hip-worn accelerometers (Evenson et al., 2013; Sallis et al., 2016). The advantage of using wrist-worn over hip-worn devices is that they can be worn continuously day and night, are water-proof, and result in higher levels of participant compliance. Although the MVPA metric we computed is not directly comparable with existing studies, it was considered appropriate for the assessment of patterns across the range of environmental characteristics and physical activity outcomes in this study.

**Self-reported physical activity**

Self-reported physical activity data were collected from a touchscreen questionnaire completed at an assessment center. The full questions used are available online (UK Biobank, 2006). Firstly, questions asked about moderate and vigorous physical activity, and total walking, similar to those used in the short form of the International Physical Activity Questionnaire (IPAQ) (The IPAQ Group, 2015).

The frequency of MVPA and total walking were captured in the following questions and information on duration was collected from participants who indicated that they did 10 minutes or more of the associated activity type.

For moderate physical activities

*"In a typical WEEK, on how many days did you do 10 minutes or more of moderate physical activities like carrying light loads, cycling at normal pace? (Do not include walking)"*

*"How many minutes did you usually spend doing moderate activities on a typical DAY?"*

For vigorous physical activities

*"In a typical WEEK, how many days did you do 10 minutes or more of vigorous physical activity? (These are activities that make you sweat or breathe hard such as fast cycling, aerobics, heavy lifting)"*

*"How many minutes did you usually spend doing vigorous activities on a typical DAY?"*

For walking

*"In a typical WEEK, on how many days did you walk for at least 10 minutes at a time? (Include walking that you do at work, travelling to and from work, and for sport or leisure)"*

*"How many minutes did you usually spend walking on a typical DAY?"*

Walking for pleasure

This information was asked in a battery of questions about leisure time activities. Participants were asked if they had spent any time walking for pleasure, not as a means of transport, within the last 4 weeks alongside a list of other activities such as sports, other exercises (such as swimming, cycling, keep fit), light DIY or heavy DIY. Those who responded positively to any of the activities were prompted to report the duration of activity using the available options.

*"How many times in the last 4 weeks did you go walking for pleasure?"*

*Once in the last 4 weeks*

*2-3 times in the last 4 weeks*

*Once a week*

*2-3 times a week*

*4-5 times a week*

*Every day*

*"Each time you went walking for pleasure, about how long did you spend doing it?"*

*Less than 15 minutes*

*Between 15 and 30 minutes*

*Between 30 minutes and 1 hour*

*Between 1 and 1.5 hours*

*Between 1.5 and 2 hours*

*Between 2 and 3 hours*

*Over 3 hours*

For all self-reported measures of activity, information on frequencies and durations were used to compute weekly time spent engaging in these activities (number of days x reported duration). Participants who did not report a frequency but a duration or a frequency but not a duration were assigned to the median frequency or duration for that activity based on responses from other participants in the sample. To calculate minutes spent in moderate-to-vigorous activity (MVPA), the weekly time spent in both moderate activity and vigorous activity were generated and summed.

For the categorical responses for frequency, these were converted to the frequency per week (i.e. those reporting engaging in activity ‘once a week’ were assigned a frequency of ‘1’ and those reporting activity ‘every day’ were assigned ‘7’). For duration in minutes, activities were assigned to the median of that category (i.e. ‘between 30 minutes and 1 hour’ was assigned ‘45 minutes’. Those responding ‘less than 15 minutes’ and ‘over 3 hours’ were assigned 7.5 minutes and 210 minutes respectively). These assignments match the ones used in the processing of self-reported physical activity from several large cohorts including EPIC-Norfolk (Wareham et al., 2002).

**Environmental variables**

Several other publications provide a detailed description of the processes used to derive data in the UK Biobank Urban Morphometric Platform (UKBUMP).(Sarkar, 2017; Sarkar et al., 2015, 2014) Briefly, participant's dwelling addresses were geocoded to building footprints and neighborhoods were defined within street network and circular buffers centered on these locations in ArcGIS12 Network Analyst. Data on building-level land uses and street networks were sourced from UK Ordnance Survey AddressBase Premium and MasterMap Integrated Transport Network databases. The UKBUMP employed a standard land use classification scheme of the Ordnance Survey AddressBase Premium database and has land use intensities of over 200 health promoting/inhibiting destinations within the defined neighborhood, as well as information on street connectivity, greenness, terrain and wider environmental characteristics pertaining to context.

For the purposes of this analysis, we chose variables which were conceptually and most plausibility related to physical activity and had support in the literature (McCormack and Shiell, 2011; Saelens et al., 2003; Van Holle et al., 2012). We selected 15 variables which were grouped into five facets (spaces for physical activity, walkability, disturbance, natural environment, and the sociodemographic environment) based on theme and their influence on different types of activity. Facets ranged from microscale environments such as facilities designed for physical activity, which are considered to encourage specific types of activity, to macroscale environments such as urban-rural status, which may affect levels of activity more generally. The hypothesized pathways between the environmental variables and physical activity outcomes are detailed in Figure S1.

**
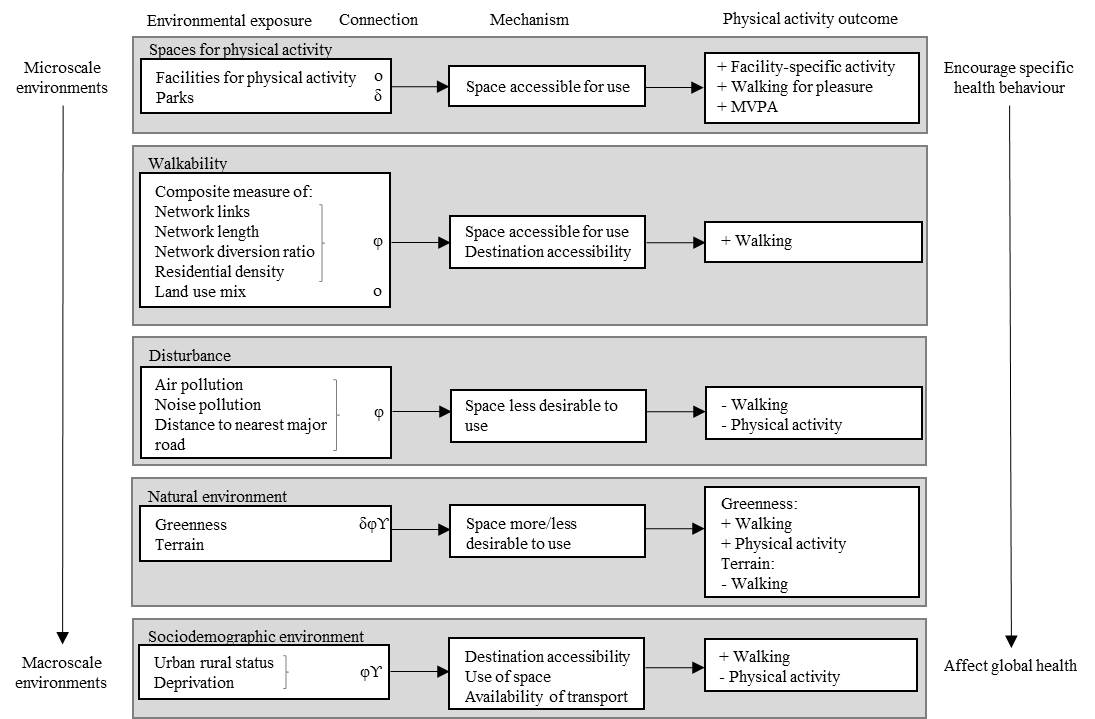
Figure S1: Hypothesized pathways between environmental characteristics and physical activity**

Exploratory analyses based on the distribution of the environmental data, previous studies, and recommended levels of pollution were performed prior to the main analysis to identify suitable cut points for each environmental factor.

**Spaces for physical activity**

Land use feature data were used to identify i) facilities designed for physical activity to take place in and ii) public parks. Features included in the classification are detailed in Table S1. Given that relatively few participants had these facilities around their home, a simple binary classification was used where neighborhoods were categorized as having access to spaces for physical activity or not. A sensitivity analysis was also performed to include facilities where activity may also take place but have not been designed purposefully for this.

**Table S1: Features included as spaces for physical activity**

|  | Included for main analysis | Included for sensitivity analysis |
| --- | --- | --- |
| i) Facilities for physical activity^a^ |  |  |
| Indoor/outdoor leisure center | ✓ | ✓ |
| Bowls facility | ✓ | ✓ |
| Cricket facility | ✓ | ✓ |
| Swimming facility | ✓ | ✓ |
| Equestrian facility | ✓ | ✓ |
| Football facility | ✓ | ✓ |
| Golf facility | ✓ | ✓ |
| Leisure/sports center | ✓ | ✓ |
| Racquet sports facility | ✓ | ✓ |
| Playing field | ✓ | ✓ |
| Recreation ground | ✓ | ✓ |
| Rugby facility | ✓ | ✓ |
| Tenpin bowling | ✓ | ✓ |
| Water sports facility | ✓ | ✓ |
| Public hall/Community facility |  | ✓ |
| Church hall |  | ✓ |
| Private social club |  | ✓ |
| Arena/stadium |  | ✓ |
|  |  |  |
| ii) Public parks |  |  |
| Park | ✓ | ✓ |
| Public park/garden | ✓ | ✓ |
| Open space |  | ✓ |
| Public open space/nature reserve |  | ✓ |

^a^Skate parks and winter sports facilities were not included as data for these features were only available for a limited portion of the sample (n=20,152 participants)

**Walkability**

For the main analysis, a composite score for walkability was derived, based on measures of street connectivity, residential density and land use mix (Creatore et al., 2016; Hajna et al., 2016; Rundle et al., 2015; Van Cauwenberg et al., 2016). The separate components are described in Table S2.

**Table S2: Description of objectively-measured walkability component variables**

|  | Variable | Description | Spatial scale  *Buffer type* | Data source, *Year* | Classification |
| --- | --- | --- | --- | --- | --- |
|  | **Walkability components** | | | | |
| Street connectivity | **Network links** | Total number of network links where a link is a street joining a junction or dead-end to a junction or dead-end | 1.2km^a^  *network* | UK OS ITN, *2010* | Quartile |
|  | **Network length** | Total length of links | 1.2km^a^  *network* | UK OS ITN, *2010* | Quartile |
|  | **Network diversion ratio** | Mean difference between crow-flight path and actual path for all links | 1.2km^a^  *network* | UK OS ITN, *2010* | Quartile |
|  | **Residential density** | Total number of residential addresses divided by total neighborhood area (no. features/square km) | 1km  *network* | UK OS AddressBase Premium point data, *2013* | Quartile |
|  | **Land use mix** | Proportion of land use squared and summed | 1km  *network* | UK OS AddressBase Premium point data, *2013* | Quartile |

^a^0.4km distance used for sensitivity analyses to investigate the effects of smaller neighborhood measures

Land use density data were available as the number of features per square kilometer. To measure land use mix, features considered to be walkable destinations were grouped into five categories: residential, retail, office, community, and recreational space based on literature, locale, and available data (Christian et al., 2011; Frank et al., 2006, 2005; Stockton et al., 2016). A land use mix score was created using the Herfindahl Hirschman Index (HHI), as used in similar studies (Arvidsson et al., 2012; Carver et al., 2014; Panter et al., 2010; Sundquist et al., 2011). The HHI (Equation S1) was considered an appropriate calculation of mix as the density measure of the data refers to the number of features, rather than the proportion of land cover. It is unlikely that there will be an equal distribution of residential features to retail features, for example, which would score highly in an entropy formula. Instead, the HHI assesses the range of land uses, with a greater number of categories per square kilometer scoring better than an equal distribution of fewer categories. HHI scores ranged from zero to 10,000 (100²) where a high score indicates a low level of land use mix.

**Equation S1: Σ(p_i_^2^)**

*p represents the proportion of features devoted to a specific land use (i) per square km of 1km network buffer*

*p is calculated by dividing the number of features in land use (i) by the total number of features of all present land uses per square km of 1km network buffer*

All network measures were divided into deciles and summed to create a street connectivity score out of 30. Z scores were generated for the combined street connectivity score, residential density, and land use mix, then summed to create a walkability score for each participant. A higher score indicated greater walkability. We also investigated the components separately and in sensitivity analysis (Creatore et al., 2016; Hajna et al., 2016; Rundle et al., 2015; Van Cauwenberg et al., 2016).

**Disturbance of the environment**

**Air pollution**

Air pollutants have been measured at 36 sample areas across Europe and modelled using a land use regression (LUR) model (Beelen et al., 2013). The LUR model accounts for predictors of air pollutants including land use, traffic, and geographic characteristics and is used to estimate outdoor air pollution at participant’s addresses (ESCAPE, 2010). Annual average concentrations of particulate matter with aerodynamic diameter ≤ 2.5 μm (PM_2.5_), nitrogen dioxide (NO_2_), and nitrogen oxides (NO_X_) are available as continuous measures within the UK Biobank dataset. Given the data available, we selected the variables to include based on a combination of preliminary analysis and our a priori conceptual rationale.

Based on the hypothesis that low air quality deters use of greenspace and the neighborhood in general, people’s perceptions of the neighborhood may impact their choice to be active. Measures of pollutants where traffic is the main source (such as NO_X_) may be important as people may avoid main or busy roads which have high volumes of traffic. Using PM_2.5_ which is the most harmful to health (GBD 2015 Risk Factors Collaborators, 2016) may be problematic as these pollutants may be difficult for people to perceive (Dons et al., 2017).

To avoid issues of collinearity and help in the decision about which measure to carry forward, we examined the univariate associations between each pollutant and our outcomes, and between pollutants. We found that NO_X_ showed the strongest and most consistent results with all physical activity outcomes (high concentrations associated with lower levels of activity). NO_X_ has been used in other epidemiological studies (Hankey et al., 2012; Nafstad et al., 2004) and an additional data site in the UK was used to generate the NO_X_ model than PM_2.5_ (Beelen et al., 2013). We therefore chose to use NOx as an indicator of air pollution. As data in UK Biobank are skewed towards lower levels of air pollution it was not sensible to use recommended levels of air quality to classify the data (Krzyzanowski and Cohen, 2008). Instead, measures of NO_X_ were dichotomized based on the median. We also included distance to the nearest major road in the models to capture the further impact of traffic volumes.

**Noise pollution**

Levels of noise pollution have been mapped based on variables including road traffic, railway traffic and industrial noise sources in Europe (Kephalopoulos et al., 2012). A measure of average daytime sound was chosen for analysis as it was assumed that most physical activity in the neighborhood would take place during the day. Levels of noise pollution were dichotomized based on the median for analysis.

**Natural environment**

**Terrain**

We hypothesis that hilly environments may not be conducive to activities such as walking. Based on the distribution of the data, we categorize terrain into the least and most hilly environments based on the median of the data.

**Greenness**

Normalized deviation index (NDVI) was calculated based on 0.5cm by 0.5cm resolution color infrared (CIR) imagery (Sarkar, 2017). The images were collected during summertime across the baseline phase of the UK Biobank study (2006-2010) and values were averaged to calculate mean NDVI to minimize temporal misclassification. Measures of greenness were classified for all participants in the same way, reducing confounding by seasonal variation.

**Sociodemographic environment**

**Urban-rural status**

Seventeen categories for urban-rural status were provided in the UKBUMP dataset based on country and home area population density. For the purposes of analysis, these categories were collapsed into three groups: urban, town and fringe, and rural.

**Statistical analysis**

Environmental data were available for participants from all assessment centers except Stockport where the pilot study was conducted. We compared both the full and the potential sample (those with environmental data) with the final analytic samples (those with either recorded or reported activity) to investigate attrition through the exclusion process of our study.

To test for collinearity between environmental variables, we examined the correlation between them and where correlations were greater than 0.5, we used the variable most strongly related to physical activity. Following the regression analyses, we looked for differences in directions of association and significance between Model 0 and Model 1 to check whether multicollinearity was driving associations seen in Model 1.

All analyses were conducted using STATA/SE 14.1.

**References**

Arvidsson, D., Kawakami, N., Ohlsson, H., Sundquist, K., 2012. Physical activity and concordance between objective and perceived walkability. Med. Sci. Sports Exerc. 44, 280–287. https://doi.org/10.1249/MSS.0b013e31822a9289

Beelen, R., Hoek, G., Vienneau, D., Eeftens, M., Dimakopoulou, K., Pedeli, X., Tsai, M.Y., Künzli, N., Schikowski, T., Marcon, A., Eriksen, K.T., Raaschou-Nielsen, O., Stephanou, E., Patelarou, E., Lanki, T., Yli-Tuomi, T., Declercq, C., Falq, G., Stempfelet, M., Birk, M., Cyrys, J., von Klot, S., Nádor, G., Varró, M.J., Dedele, A., Gražulevičiene, R., Mölter, A., Lindley, S., Madsen, C., Cesaroni, G., Ranzi, A., Badaloni, C., Hoffmann, B., Nonnemacher, M., Krämer, U., Kuhlbusch, T., Cirach, M., de Nazelle, A., Nieuwenhuijsen, M., Bellander, T., Korek, M., Olsson, D., Strömgren, M., Dons, E., Jerrett, M., Fischer, P., Wang, M., Brunekreef, B., de Hoogh, K., 2013. Development of NO2 and NOx land use regression models for estimating air pollution exposure in 36 study areas in Europe - The ESCAPE project. Atmos. Environ. 72, 10–23. https://doi.org/10.1016/j.atmosenv.2013.02.037

Carver, A., Panter, J.R., Jones, A.P., van Sluijs, E.M.F., 2014. Independent mobility on the journey to school: A joint cross-sectional and prospective exploration of social and physical environmental influences. J. Transp. Heal. 1, 25–32. https://doi.org/10.1016/j.jth.2013.12.003

Christian, H.E., Bull, F.C., Middleton, N.J., Knuiman, M.W., Divitini, M.L., Hooper, P., Amarasinghe, A., Giles-Corti, B., 2011. How important is the land use mix measure in understanding walking behaviour? Results from the RESIDE study. Int. J. Behav. Nutr. Phys. Act. 8, 55. https://doi.org/10.1186/1479-5868-8-55

Creatore, M.I., Glazier, R.H., Moineddin, R., Fazli, G.S., Johns, A., Gozdyra, P., Matheson, F.I., Kaufman-Shriqui, V., Rosella, L.C., Manuel, D.G., Booth, G.L., 2016. Association of Neighborhood Walkability With Change in Overweight, Obesity, and Diabetes. Jama 315, 2211.

Doherty, A., Jackson, D., Hammerla, N., Plötz, T., Olivier, P., Granat, M., White, T., van Hees, V., Trenell, M., Owen, C., Preece, S., Gillions, R., Sheard, S., Peakman, T., Brage, S., Wareham, N., 2017. Large Scale Population Assessment of Physical Activity Using Wrist Worn Accelerometers: The UK Biobank Study. PLoS One 12. https://doi.org/10.1371/journal.pone.0169649

Dons, E., Laeremans, M., Boig, E.A., Avila-Palencia, I., Brand, C., Cole-Hunter, T., de Nazelle, A., Gaupp-Berghausen, M., Gerike, R., Götschi, T., Mueller, N., Nieuwenhuijsen, M., Orjuela, J.P., Raser, E., Rojas-Rueda, D., Standaert, A., Stigell, E., Panis, L.I., 2017. NO2 but Not PM2.5 at the Home Address is Associated with Concern over Health Effects of Air Pollution. J. Transp. Heal. 5, S84–S114. https://doi.org/10.1016/J.JTH.2017.05.251

ESCAPE, 2010. ESCAPE Exposure Assessment Manual: version July 2010 [WWW Document]. URL http://www.escapeproject.eu/manuals/ESCAPE_Exposure-manualv9.pdf

Evenson, K.R., Wen, F., Hillier, A., Cohen, D.A., 2013. Assessing the contribution of parks to physical activity using global positioning system and accelerometry. Med. Sci. Sports Exerc. 45, 1981–1987. https://doi.org/10.1249/MSS.0b013e318293330e

Frank, L.D., Sallis, J.F., Conway, T.L., Chapman, J.E., Saelens, B.E., Bachman, W., 2006. Many Pathways from Land Use to Health: Associations between Neighborhood Walkability and Active Transportation, Body Mass Index, and Air Quality. J. Am. Plan. Assoc. 72, 75–87. https://doi.org/10.1080/01944360608976725

Frank, L.D., Schmid, T.L., Sallis, J.F., Chapman, J., Saelens, B.E., 2005. Linking objectively measured physical activity with objectively measured urban form: Findings from SMARTRAQ. Am. J. Prev. Med. 28, 117–125. https://doi.org/10.1016/j.amepre.2004.11.001

GBD 2015 Risk Factors Collaborators, 2016. Global, regional, and national comparative risk assessment of 79 behavioural, environmental and occupational, and metabolic risks or clusters of risks, 1990–2015: a systematic analysis for the Global Burden of Disease Study 2015. Lancet 388, 1659–1724. https://doi.org/https://doi.org/10.1016/S0140-6736(16)31679-8

Hajna, S., Kestens, Y., Daskalopoulou, S.S., Joseph, L., Thierry, B., Sherman, M., Trudeau, L., Rabasa-Lhoret, R., Meissner, L., Bacon, S.L., Gauvin, L., Ross, N.A., Dasgupta, K., Diabetes, GPS, and W.S.G., 2016. Neighbourhood walkability and home neighbourhood-based physical activity: an observational study of adults with type 2 diabetes. BMC Public Health 16, 957. https://doi.org/10.1186/s12889-016-3603-y

Hankey, S., Marshall, J.D., Brauer, M., 2012. Health impacts of the built environment: Within-urban variability in physical inactivity, air pollution, and ischemic heart disease mortality. Environ. Health Perspect. 120, 247–253. https://doi.org/10.1289/ehp.1103806

Kephalopoulos, S., Paviotti, M., Anfosso‐Lédée, F., 2012. Common noise assessment methods in EU: CNOSSOS-EU, Publications Office of the European Union. https://doi.org/10.2788/31776

Krzyzanowski, M., Cohen, A., 2008. Update of WHO air quality guidelines. Air Qual. Atmos. Heal. 1, 7–13. https://doi.org/10.1007/s11869-008-0008-9

McCormack, G.R., Shiell, A., 2011. In search of causality: a systematic review of the relationship between the built environment and physical activity among adults. Int. J. Behav. Nutr. Phys. Act. 8, 125. https://doi.org/10.1186/1479-5868-8-125

Nafstad, P., Haheim, L.L., Wisloff, T., Gram, F., Oftedal, B., Holme, I., Hjermann, I., Leren, P., 2004. Urban air pollution and mortality in a cohort of norwegian men. Env. Heal. Perspect 112, 610–615. https://doi.org/10.1289/ehp.6684

Panter, J.R., Jones, A.P., Van Sluijs, E.M.F., Griffin, S.J., 2010. Neighborhood, Route, and School Environments and Children’s Active Commuting. Am. J. Prev. Med. 38, 268–278. https://doi.org/10.1016/j.amepre.2009.10.040

Rundle, A.G., Sheehan, D.M., Quinn, J.W., Bartley, K., Eisenhower, D., Bader, M.M.D., Lovasi, G.S., Neckerman, K.M., 2015. Using GPS Data to Study Neighborhood Walkability and Physical Activity. Am. J. Prev. Med. 50, e65–e72. https://doi.org/10.1016/j.amepre.2015.07.033

Saelens, B.E., Sallis, J.F., Frank, L.D., 2003. Environmental correlates of walking and cycling: findings from the transportation, urban design, and planning literatures. Ann. Behav. Med. a Publ. Soc. Behav. Med. 25, 80–91.

Sallis, J.F., Cerin, E., Conway, T.L., Adams, M.A., Frank, L.D., Pratt, M., Salvo, D., Schipperijn, J., Smith, G., Cain, K.L., Davey, R., Kerr, J., Lai, P.C., Mitáš, J., Reis, R., Sarmiento, O.L., Schofield, G., Troelsen, J., Van Dyck, D., De Bourdeaudhuij, I., Owen, N., 2016. Physical activity in relation to urban environments in 14 cities worldwide: A cross-sectional study. Lancet 387, 2207–2217. https://doi.org/10.1016/S0140-6736(15)01284-2

Sarkar, C., 2017. Residential greenness and adiposity: Findings from the UK Biobank. Environ. Int. 106, 1–10. https://doi.org/10.1016/j.envint.2017.05.016

Sarkar, C., Gallacher, J., Webster, C., 2014. Morphometric Analysis of the Built Environment in UK Biobank: Data Analyses and Specification Manual [WWW Document]. URL https://biobank.ctsu.ox.ac.uk/crystal/docs/ard-1195_UKBBuiltEnvWales.pdf (accessed 5.17.17).

Sarkar, C., Webster, C., Gallacher, J., 2015. UK Biobank Urban Morphometric Platform (UKBUMP) – a nationwide resource for evidence-based healthy city planning and public health interventions. Ann. GIS 21, 135–148. https://doi.org/10.1080/19475683.2015.1027791

Stockton, J.C., Duke-Williams, O., Stamatakis, E., Mindell, J.S., Brunner, E.J., Shelton, N.J., 2016. Development of a novel walkability index for London, United Kingdom: cross-sectional application to the Whitehall II Study. BMC Public Health 16, 416.

Sundquist, K., Eriksson, U., Kawakami, N., Skog, L., Ohlsson, H., Arvidsson, D., 2011. Neighborhood walkability, physical activity, and walking behavior: The Swedish Neighborhood and Physical Activity (SNAP) study. Soc. Sci. Med. 72, 1266–1273. https://doi.org/10.1016/j.socscimed.2011.03.004

The IPAQ Group, 2015. International Physical Activity Questionnaire [WWW Document]. URL https://sites.google.com/site/theipaq/home

UK Biobank, 2006. UK Biobank Touchscreen Questionnaire: final version [WWW Document]. URL https://www.ukbiobank.ac.uk/wp-content/uploads/2011/06/Touch_screen_questionnaire.pdf?phpMyAdmin=trmKQlYdjjnQIgJ%2CfAzikMhEnx6 (accessed 5.17.17).

Van Cauwenberg, J., Van Holle, V., De Bourdeaudhuij, I., Van Dyck, D., Deforche, B., 2016. Neighborhood walkability and health outcomes among older adults: The mediating role of physical activity. Health Place 37, 16–25. https://doi.org/10.1016/j.healthplace.2015.11.003

Van Holle, V., Deforche, B., Van Cauwenberg, J., Goubert, L., Maes, L., Van de Weghe, N., De Bourdeaudhuij, I., 2012. Relationship between the physical environment and different domains of physical activity in European adults: a systematic review. BMC Public Health 12, 807. https://doi.org/10.1186/1471-2458-12-807

Wareham, N.J., Jakes, R.W., Rennie, K.L., Mitchell, J., Hennings, S., Day, N.E., 2002. Validity and repeatability of the EPIC-Norfolk Physical Activity Questionnaire. Int. J. Epidemiol. 31, 168–174. https://doi.org/10.1093/ije/31.1.168

White, T., Westgate, K., Wareham, N.J., Brage, S., 2016. Estimation of physical activity energy expenditure during free-living from wrist accelerometry in UK adults. PLoS One 11. https://doi.org/10.1371/journal.pone.0167472
