# Supplementary material for: Characteristics of the environment and physical activity in midlife: Findings from UK Biobank
Source: Prev Med. 2019 Jan;118:150–8. doi: 10.1016/j.ypmed.2018.10.024 (PMC6344227; doi:10.1016/j.ypmed.2018.10.024)
Supplement: Supplementary file 2 — Supplemental File 2 Additional results [file mmc2.docx]

Supplemental File 2: Additional results

*Table S1: Adjusted cross-sectional associations between environmental characteristics and physical activity outcomes (Model 1)*

|  |  | Recorded measures | | | Reported measures | | | | | |
| --- | --- | --- | --- | --- | --- | --- | --- | --- | --- | --- |
|  |  | **Mean acceleration** | **MVPA** | | **MVPA** | | **Total walking** | | **Walking for pleasure** | |
|  |  |  | Middle tertile | Upper tertile | Middle tertile | Upper tertile | Middle tertile | Upper tertile | Middle tertile | Upper tertile |
|  |  | β (95% CI) | RRR (95% CI) | RRR (95% CI) | RRR (95% CI) | RRR (95% CI) | RRR (95% CI) | RRR (95% CI) | RRR (95% CI) | RRR (95% CI) |
| Spaces for PA | **Facilities for PA** (ref: none) | † | n.s | † | *n.i* | *n.i* | * | * | n.s | * |
|  | One or more | 0.19 (0.05, 0.33) | 1.04 (1.00, 1.09) | 1.06 (1.01, 1.11) |  |  | 1.03 (1.01, 1.05) | 1.03 (1.01, 1.05) | 1.01 (0.99, 1.03) | 1.03 (1.01, 1.05) |
|  | **Parks** (ref: none) | *n.i* | n.s | n.s | *n.i* | *n.i* | n.s | n.s | † | n.s |
|  | One or more |  | 0.99 (0.95, 1.05) | 1.01 (0.96, 1.06) |  |  | 1.00 (0.98, 1.03) | 0.99 (0.97, 1.02) | 1.02 (1.00, 1.04) | 1.01 (0.99, 1.03) |
| Walk-ability | **Walkability** (ref: lowest) | ** | ** | ** | ** | ** | ** | ** | ** | n.s |
|  | Q2 | 0.04 (-0.15, 0.22) | 1.01 (0.96, 1.07) | 1.03 (0.98, 1.10) | 1.01 (0.98, 1.03) | 1.03 (1.01, 1.06) | 1.01 (0.98, 1.03) | 1.04 (1.02, 1.07) | 0.98 (0.95, 1.00) | 0.98 (0.95, 1.00) |
|  | Q3 | 0.12 (-0.08, 0.32) | 1.06 (1.00, 1.13) | 1.08 (1.01, 1.15) | 1.02 (1.00, 1.05) | 1.07 (1.04, 1.10) | 1.05 (1.02, 1.08) | 1.07 (1.04, 1.10) | 0.97 (0.94, 1.00) | 0.96 (0.93, 0.99) |
|  | Q4 | 0.45 (0.23, 0.67) | 1.15 (1.07, 1.23) | 1.28 (1.20, 1.38) | 1.09 (1.06, 1.12) | 1.12 (1.09, 1.15) | 1.16 (1.13, 1.20) | 1.14 (1.10, 1.17) | 1.07 (1.04, 1.11) | 1.03 (1.00, 1.06) |
| Disturbance | **NO_X_** (ref: lowest) | ** | † | ** | ** | ** | n.s | * | * | ** |
|  | Highest | -0.57 (-0.84, -0.30) | 0.92 (0.85, 1.00) | 0.86 (0.79, 0.94) | 0.93 (0.89, 0.96) | 0.84 (0.81, 0.88) | 1.00 (0.97, 1.04) | 0.93 (0.90, 0.97) | 0.93 (0.90, 0.97) | 0.85 (0.81, 0.88) |
|  | **Noise pollution** (ref: lowest) | *n.i* | *n.i* | *n.i* | *n.i* | *n.i* | n.s | † | n.s | n.s |
|  | Highest |  |  |  |  |  | 1.01 (0.99, 1.03) | 1.02 (1.00, 1.04) | 1.00 (0.98, 1.01) | 1.02 (1.00, 1.03) |
|  | **Distance to major road** (ref: closest) | *n.i* | *n.i* | *n.i* | *n.i* | *n.i* | n.s | n.s | *n.i* | *n.i* |
|  | Furthest |  |  |  |  |  | 1.00 (0.99, 1.01) | 0.99 (0.98, 1.00) |  |  |
| Natural environment | **Terrain** (ref: mean slope <3°) | *n.i* | *n.i* | *n.i* | *n.i* | *n.i* | n.s | † | ** | ** |
|  | Mean slope ≥3° |  |  |  |  |  | 0.99 (0.98, 1.01) | 1.02 (1.01, 1.04) | 1.05 (1.04, 1.07) | 1.08 (1.06, 1.10) |
|  | **Greenness** (ref: least) | *n.i* | *n.i* | *n.i* | n.s | ** | ** | ** | n.s | ** |
|  | Q2 |  |  |  | 1.04 (1.01, 1.06) | 1.10 (1.07, 1.12) | 1.03 (1.01, 1.06) | 1.06 (1.03, 1.08) | 1.01 (0.99, 1.04) | 1.02 (1.00, 1.05) |
|  | Q3 |  |  |  | 1.02 (1.00, 1.04) | 1.09 (1.06, 1.11) | 1.05 (1.02, 1.07) | 1.06 (1.04, 1.09) | 1.01 (0.99, 1.04) | 1.02 (1.00, 1.05) |
|  | Q4 |  |  |  | 1.00 (0.98, 1.03) | 1.08 (1.05, 1.10) | 1.09 (1.06, 1.12) | 1.14 (1.11, 1.17) | 1.00 (0.98, 1.03) | 1.08 (1.05, 1.11) |
| Sociodemographic | **Urban-rural status** (ref: urban) | ** | n.s | ** | ** | ** | n.s | ** | ** | ** |
|  | Fringe | 0.30 (0.05, 0.54) | 0.98 (0.91, 1.06) | 1.07 (0.99, 1.15) | 1.05 (1.01, 1.08) | 1.08 (1.05, 1.12) | 1.04 (1.00, 1.07) | 1.09 (1.05, 1.13) | 1.16 (1.12, 1.21) | 1.26 (1.22, 1.30) |
|  | Rural | 0.83 (0.53, 1.12) | 1.00 (0.92, 1.10) | 1.18 (1.07, 1.30) | 1.09 (1.04, 1.14) | 1.21 (1.16, 1.27) | 1.01 (0.97, 1.06) | 1.12 (1.07, 1.17) | 1.11 (1.06, 1.17) | 1.25 (1.20, 1.31) |
|  | **Area-level deprivation** (ref: least deprived) | ** | ** | ** | ** | † | † | ** | ** | ** |
|  | Q2 | -0.06 (-0.24, 0.13) | 0.96 (0.90, 1.01) | 0.96 (0.90, 1.01) | 1.00 (0.97, 1.03) | 1.00 (0.97, 1.03) | 1.03 (1.00, 1.05) | 1.06 (1.03, 1.09) | 0.98 (0.95, 1.00) | 0.99 (0.96, 1.01) |
|  | Q3 | -0.16 (-0.36, 0.03) | 0.90 (0.85, 0.95) | 0.90 (0.85, 0.96) | 0.98 (0.95, 1.01) | 1.01 (0.98, 1.04) | 1.02 (0.99, 1.05) | 1.07 (1.04, 1.09) | 0.91 (0.88, 0.93) | 0.90 (0.88, 0.92) |
|  | Q4 | -0.42 (-0.63, -0.22) | 0.86 (0.81, 0.92) | 0.87 (0.81, 0.92) | 0.95 (0.92, 0.97) | 1.00 (0.97, 1.02) | 1.03 (1.00, 1.06) | 1.09 (1.06, 1.12) | 0.85 (0.83, 0.88) | 0.82 (0.80, 0.84) |
|  | Most deprived | -0.82 (-1.06, -0.59) | 0.80 (0.74, 0.86) | 0.80 (0.74, 0.86) | 0.89 (0.86, 0.92) | 0.96 (0.93, 0.99) | 1.03 (1.00, 1.06) | 1.09 (1.06, 1.13) | 0.77 (0.74, 0.79) | 0.74 (0.72, 0.76) |

Model adjusted for age, sex, ethnicity, education, income, car ownership, assessment center, housing tenure, employment status, children in household, urban-rural status, area-level deprivation plus significant environmental characteristics from univariate analyses (Model 0). Walkability components have been substituted for walkability summary score

**p<0.001 *p<0.01 †p<0.05 indicates test for trend. β – regression coefficient; RRR – relative risk ratio; CI – confidence interval; *n.i* – not included in model

*Table S2: Adjusted cross-sectional associations between environmental characteristics, including walkability components as separate variables, and physical activity outcomes*

|  |  | Recorded measures | | | Reported measures | | | | | |
| --- | --- | --- | --- | --- | --- | --- | --- | --- | --- | --- |
|  |  | **Mean acceleration** | **MVPA** | | **MVPA** | | **Total walking** | | **Walking for pleasure** | |
|  |  |  | Middle tertile | Upper tertile | Middle tertile | Upper tertile | Middle tertile | Upper tertile | Middle tertile | Upper tertile |
|  |  | β (95% CI) | RRR (95% CI) | RRR (95% CI) | RRR (95% CI) | RRR (95% CI) | RRR (95% CI) | RRR (95% CI) | RRR (95% CI) | RRR (95% CI) |
| Spaces for PA | **Facilities for PA** (ref: none) | † | n.s | † | *n.i* | *n.i* | * | † | n.s | n.s |
|  | One or more | 0.16 (0.01, 0.30) | 1.04 (0.99, 1.08) | 1.05 (1.01, 1.10) |  |  | 1.03 (1.01, 1.05) | 1.03 (1.01, 1.05) | 1.00 (0.98, 1.02) | 1.02 (1.00, 1.04) |
|  | **Parks** (ref: none) | *n.i* | n.s | n.s | *n.i* | *n.i* | n.s | n.s | n.s | n.s |
|  | One or more |  | 0.99 (0.94, 1.04) | 1.00 (0.95, 1.06) |  |  | 1.00 (0.98, 1.03) | 0.99 (0.97, 1.01) | 1.02 (0.99, 1.04) | 1.00 (0.98, 1.03) |
| Walkability | **Street connectivity** (ref: least) | * | n.s | ** | * | † | n.s | n.s | ** | * |
|  | Q2 | 0.06 (-0.12, 0.24) | 1.03 (0.97, 1.09) | 1.06 (1.00, 1.12) | 1.02 (1.00, 1.05) | 1.03 (1.00, 1.05) | 1.02 (0.99, 1.04) | 1.01 (0.99, 1.03) | 1.01 (0.98, 1.03) | 1.00 (0.98, 1.03) |
|  | Q3 | 0.11 (-0.07, 0.29) | 1.04 (0.98, 1.10) | 1.10 (1.03, 1.16) | 1.03 (1.00, 1.06) | 1.06 (1.03, 1.09) | 1.01 (0.98, 1.04) | 1.01 (0.99, 1.04) | 1.03 (1.00, 1.05) | 1.03 (1.00, 1.05) |
|  | Q4 | 0.32 (0.13, 0.52) | 1.06 (0.99, 1.14) | 1.19 (1.11, 1.27) | 1.04 (1.01, 1.07) | 1.02 (0.99, 1.05) | 1.02 (0.99, 1.05) | 0.98 (0.95, 1.01) | 1.08 (1.05, 1.12) | 1.05 (1.02, 1.08) |
|  | **Dwelling density** (ref: lowest) | *n.i* | n.s | n.s | n.s | n.s | ** | * | ** | ** |
|  | Q2 |  | 1.03 (0.97, 1.09) | 0.94 (0.88, 1.00) | 0.97 (0.94, 0.99) | 0.94 (0.91, 0.96) | 1.05 (1.02, 1.07) | 1.01 (0.98, 1.03) | 0.99 (0.96, 1.01) | 0.94 (0.91, 0.96) |
|  | Q3 |  | 1.00 (0.93, 1.07) | 0.89 (0.83, 0.95) | 0.97 (0.94, 1.00) | 0.95 (0.92, 0.98) | 1.06 (1.03, 1.09) | 1.04 (1.01, 1.07) | 0.93 (0.90, 0.96) | 0.87 (0.84, 0.89) |
|  | Q4 |  | 1.08 (1.00, 1.17) | 1.03 (0.95, 1.11) | 1.02 (0.98, 1.05) | 0.99 (0.96, 1.03) | 1.15 (1.12, 1.19) | 1.05 (1.02, 1.09) | 0.94 (0.91, 0.98) | 0.85 (0.83, 0.88) |
|  | **Land use mix** (ref: lowest) | ** | * | ** | * | ** |  | ** | ** | ** |
|  | Q2 | 0.12 (-0.06, 0.30) | 1.01 (0.96, 1.07) | 1.02 (0.96, 1.08) | 1.01 (0.98, 1.03) | 1.03 (1.01, 1.06) | 1.02 (1.00, 1.05) | 1.04 (1.02, 1.07) | 1.03 (1.00, 1.05) | 1.03 (1.01, 1.06) |
|  | Q3 | 0.21 (0.03, 0.40) | 1.05 (0.99, 1.11) | 1.04 (0.98, 1.10) | 1.00 (0.98, 1.03) | 1.03 (1.00, 1.05) | 1.03 (1.00, 1.05) | 1.07 (1.04, 1.10) | 1.03 (1.00, 1.05) | 1.05 (1.02, 1.08) |
|  | Q4 | 0.34 (0.15, 0.52) | 1.08 (1.02, 1.15) | 1.11 (1.05, 1.18) | 1.03 (1.01, 1.06) | 1.08 (1.05, 1.11) | 1.10 (1.07, 1.13) | 1.16 (1.13, 1.19) | 1.08 (1.05, 1.11) | 1.14 (1.11, 1.17) |
| Disturbance | **NO_X_** (ref: lowest) | ** | † | ** | ** | ** | n.s | † | † | ** |
|  | Highest | -0.55 (-0.82, -0.28) | 0.92 (0.85, 1.00) | 0.88 (0.81, 0.96) | 0.93 (0.90, 0.97) | 0.86 (0.83, 0.90) | 1.00 (0.96, 1.04) | 0.95 (0.91, 0.99) | 0.95 (0.91, 0.99) | 0.88 (0.85, 0.91) |
|  | **Noise pollution** (ref: lowest) | *n.i* | *n.i* | *n.i* | *n.i* | *n.i* | n.s | * | n.s | n.s |
|  | Highest |  |  |  |  |  | 1.01 (1.00, 1.03) | 1.03 (1.01, 1.05) | 0.99 (0.97, 1.01) | 1.00 (0.99, 1.02) |
|  | **Distance to major road** (ref: closest) | *n.i* | *n.i* | *n.i* | *n.i* | *n.i* | n.s | † | *n.i* | *n.i* |
|  | Furthest |  |  |  |  |  | 1.00 (0.99, 1.01) | 0.99 (0.98, 1.00) |  |  |
| Natural environment | **Terrain (**ref: mean slope <3°) | ***n.i*** | *n.i* | *n.i* | *n.i* | *n.i* | **n.s** | **n.s** | ****** | ** |
|  | Mean slope ≥3° |  |  |  |  |  | 1.00 (0.98, 1.02) | 1.02 (1.00, 1.04) | 1.04 (1.03, 1.06) | 1.06 (1.04, 1.08) |
|  | **Greenness** (ref: least) | *n.i* | *n.i* | *n.i* | n.s | ** | ** | ** | n.s | ** |
|  | Q2 |  |  |  | 1.04 (1.01, 1.06) | 1.10 (1.07, 1.12) | 1.04 (1.01, 1.06) | 1.06 (1.03, 1.09) | 1.01 (0.99, 1.04) | 1.02 (1.00, 1.05) |
|  | Q3 |  |  |  | 1.02 (0.99, 1.04) | 1.09 (1.06, 1.11) | 1.04 (1.02, 1.07) | 1.07 (1.05, 1.10) | 1.02 (0.99, 1.04) | 1.04 (1.02, 1.07) |
|  | Q4 |  |  |  | 1.00 (0.98, 1.03) | 1.08 (1.06, 1.11) | 1.09 (1.06, 1.12) | 1.15 (1.12, 1.18) | 1.01 (0.99, 1.04) | 1.10 (1.07, 1.13) |

| Sociodemographic | Urban-rural status (ref: urban) | ** | n.s | * | ** | ** | n.s | ** | ** | ** |
| --- | --- | --- | --- | --- | --- | --- | --- | --- | --- | --- |
|  | Fringe | 0.25 (0.01, 0.50) | 0.97 (0.90, 1.05) | 1.03 (0.95, 1.12) | 1.04 (1.00, 1.07) | 1.05 (1.02, 1.09) | 1.05 (1.01, 1.08) | 1.07 (1.03, 1.11) | 1.14 (1.10, 1.18) | 1.20 (1.16, 1.24) |
|  | Rural | 0.76 (0.46, 1.07) | 1.00 (0.91, 1.10) | 1.12 (1.01, 1.24) | 1.07 (1.02, 1.12) | 1.16 (1.11, 1.21) | 1.02 (0.97, 1.06) | 1.08 (1.03, 1.13) | 1.08 (1.03, 1.14) | 1.16 (1.11, 1.21) |
|  | **Area-level deprivation** (ref: least) | ** | ** | ** | ** | n.s | n.s | ** | ** | ** |
|  | Q2 | -0.06 (-0.25, 0.12) | 0.96 (0.90, 1.01) | 0.96 (0.90, 1.02) | 1.00 (0.97, 1.03) | 1.00 (0.98, 1.03) | 1.02 (0.99, 1.05) | 1.06 (1.03, 1.08) | 0.98 (0.95, 1.01) | 0.99 (0.97, 1.02) |
|  | Q3 | -0.17 (-0.36, 0.03) | 0.90 (0.84, 0.95) | 0.91 (0.86, 0.97) | 0.98 (0.96, 1.01) | 1.02 (0.99, 1.05) | 1.01 (0.98, 1.04) | 1.06 (1.03, 1.09) | 0.91 (0.89, 0.94) | 0.91 (0.89, 0.94) |
|  | Q4 | -0.41 (-0.61, -0.21) | 0.86 (0.80, 0.92) | 0.87 (0.82, 0.93) | 0.95 (0.92, 0.97) | 1.01 (0.98, 1.03) | 1.02 (0.99, 1.05) | 1.09 (1.06, 1.12) | 0.86 (0.84, 0.89) | 0.84 (0.82, 0.86) |
|  | Q5 | -0.78 (-1.02, -0.55) | 0.80 (0.74, 0.86) | 0.80 (0.74, 0.87) | 0.89 (0.86, 0.92) | 0.97 (0.94, 1.01) | 1.02 (0.98, 1.05) | 1.10 (1.06, 1.14) | 0.78 (0.76, 0.81) | 0.77 (0.74, 0.79) |

Model adjusted for age, sex, ethnicity, education, income, car ownership, assessment center, housing tenure, employment status, children in household, urban-rural status, area-level deprivation plus significant environmental characteristics from univariate analyses (Model 0).

**p<0.001 *p<0.01 †p<0.05 indicates test for trend. β – regression coefficient; RRR – relative risk ratio; CI – confidence interval; *n.i* – not included in model

*Figure S1: Adjusted associations between environmental characteristics and activity outcomes
Outcome variables: Continuous data; Upper tertile; Middle tertile; 95% Confidence interval. Results of original analyses (Model 1: 1km neighborhood measures) shown in black; Results of sensitivity analyses (o.5km neighborhood measures) shown in green. White space is where variables have not been included in adjusted model
β = regression coefficient presented on linear scale; RRR = relative risk ratio presented on log scale; MVPA = moderate-to-vigorous physical activity*

**Spaces for PA**

≥1 (ref: none)

≥1 (ref: none)

**Parks**

≥1 (ref: none)

≥1 (ref: none)

**Walkability**

Q4 highest

Q3

Q2 (ref: Q1 lowest)

Q4 highest

Q3

Q2 (ref: Q1 lowest)

**Air pollution**

Highest (ref: lowest)

Highest (ref: lowest)

**Noise pollution**

Highest (ref: lowest)

Highest (ref: lowest)

**Distance to major road**

Furthest (ref: closest)

Furthest (ref: closest)

**Terrain**

Mean slope ≥3° (ref: <3°)

Mean slope ≥3° (ref: <3°)

**Greenness**

Q4 most

Q3

Q2 (ref: Q1 least)

Q4 most

Q3

Q2 (ref: Q1 least)

**Urban-rural status**

Rural

Fringe (ref: urban)

Rural

Fringe (ref: urban)

**Area-level deprivation**

Q5 most

Q4

Q3

Q2 (ref: Q1 least)

Q5 most

Q4

Q3

Q2 (ref: Q1 least)


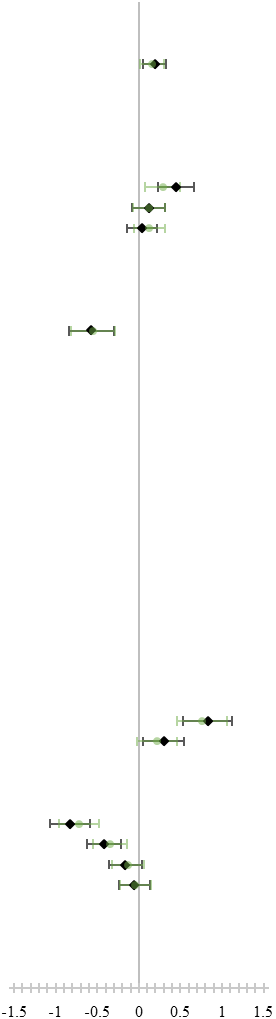

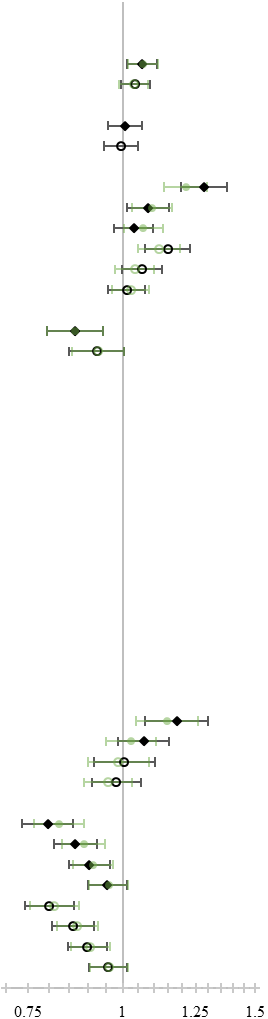

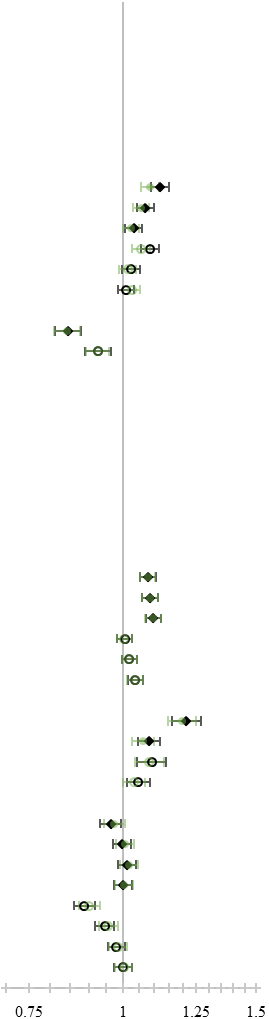

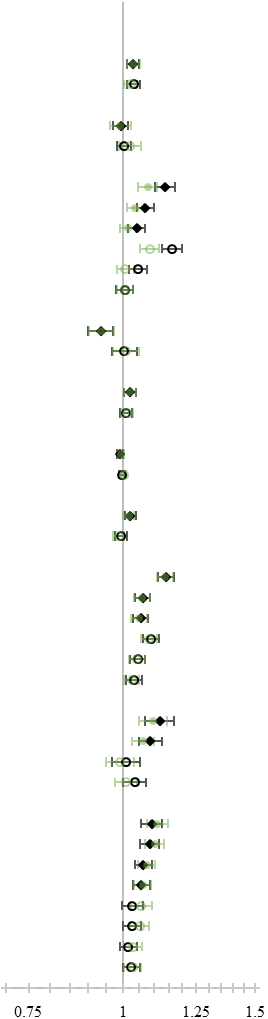

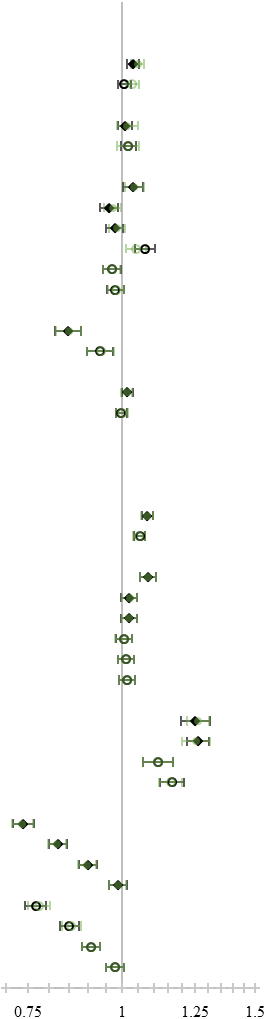


*Recorded measures Reported measures*

*Mean acceleration MVPA MVPA Total walking Walking for pleasure*

β

RRR

RRR

RRR

RRR
